# PseUdeep: RNA Pseudouridine Site Identification with Deep Learning Algorithm

**DOI:** 10.3389/fgene.2021.773882

**Published:** 2021-11-18

**Authors:** Jujuan Zhuang, Danyang Liu, Meng Lin, Wenjing Qiu, Jinyang Liu, Size Chen

**Affiliations:** ^1^ College of Science, Dalian Maritime University, Dalian, China; ^2^ Electrical and Information Engineering, Anhui University of Technology, Anhui, China; ^3^ Geneis (Beijing) Co., Ltd., Beijing, China; ^4^ Department of Oncology, The First Affiliated Hospital of Guangdong Pharmaceutical University, Guangzhou, China; ^5^ Guangdong Provincial Engineering Research Center for Esophageal Cancer Precise Therapy, The First Affiliated Hospital of Guangdong Pharmaceutical University, Guangzhou, China; ^6^ Central Laboratory, The First Affiliated Hospital of Guangdong Pharmaceutical University, Guangzhou, China

**Keywords:** RNA modification, pseudouridine site prediction, feature extraction, deep learning, capsule network

## Abstract

**Background:** Pseudouridine (Ψ) is a common ribonucleotide modification that plays a significant role in many biological processes. The identification of Ψ modification sites is of great significance for disease mechanism and biological processes research in which machine learning algorithms are desirable as the lab exploratory techniques are expensive and time-consuming.

**Results:** In this work, we propose a deep learning framework, called PseUdeep, to identify Ψ sites of three species: *H. sapiens*, *S. cerevisiae*, and *M. musculus*. In this method, three encoding methods are used to extract the features of RNA sequences, that is, one-hot encoding, K-tuple nucleotide frequency pattern, and position-specific nucleotide composition. The three feature matrices are convoluted twice and fed into the capsule neural network and bidirectional gated recurrent unit network with a self-attention mechanism for classification.

**Conclusion:** Compared with other state-of-the-art methods, our model gets the highest accuracy of the prediction on the independent testing data set S-200; the accuracy improves 12.38%, and on the independent testing data set H-200, the accuracy improves 0.68%. Moreover, the dimensions of the features we derive from the RNA sequences are only 109,109, and 119 in *H. sapiens*, *M. musculus*, and *S. cerevisiae*, which is much smaller than those used in the traditional algorithms. On evaluation via tenfold cross-validation and two independent testing data sets, PseUdeep outperforms the best traditional machine learning model available. PseUdeep source code and data sets are available at https://github.com/dan111262/PseUdeep.

## Introduction

**FIGURE 1:**
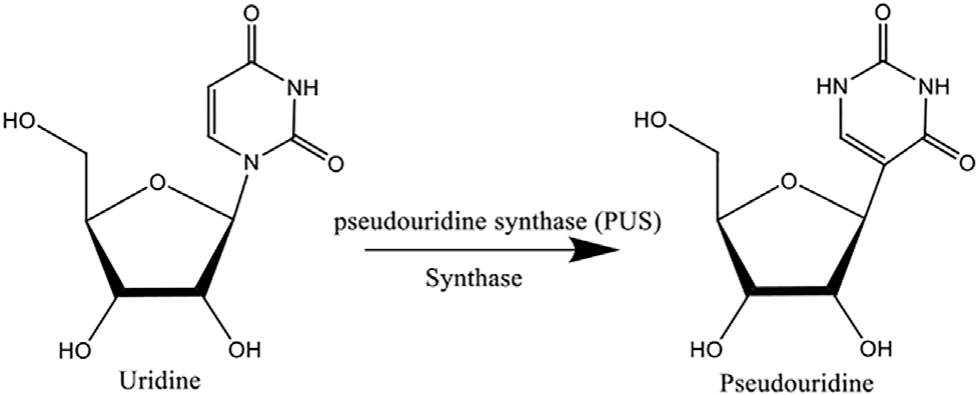
Illustration of Ψ modification. The Ψ synthase catalyzes the uridine isomer Ψ by removing the uridine residue base from its sugar and then removing the uridine isomer, rotating it 180° along the N3–C6 axis, and finally turning the base the 5-carbon and 1′-carbons of the sugar.

Pseudouridine (Ψ) is one of the most prevalent RNA modifications that occurs at the uridinebase through an isomerization reaction catalyzed by pseudouridine synthases (see Figure 1) (Bousquet-Antonelli et al., 1997; Chan and Huang, 2009; Ge and Yu, 2013; Kiss et al., 2010; Wolin, 2016; Yu and Meier, 2014). It is confirmed that Ψ modification occurs in several kinds of RNAs, such as small nuclear RNA, rRNA, tRNA, mRNA, and small nucleolar RNA (Ge and Yu, 2013). Ψ plays a significant role in many biological processes, including regulating the stability of RNA structure in tRNA and rRNA (Kierzek et al., 2014). Deficiency of Ψ might cause various diseases; the dysregulation of Ψ in mitochondrial tRNA is one of the etiologies of erythrocytic anemia and mitochondrial myopathy (Bykhovskaya et al., 2004). Moreover, the mutations of Ψ are also associated with several types of cancers, such as gastric and lung cancer (Mei et al., 2012; Carlile et al., 2014; Carlile et al., 2015; Shaheen et al., 2016; Penzo et al., 2017; Zhang et al., 2021), and Ψ is also applied in biochemical research and pharmaceuticals (C. Liu et al., 2020; Penzo et al., 2017; J. Yang et al., 2020). Undoubtedly, the identification of Ψ modification sites would be of great benefit for disease mechanism and biological processes research.

Although accurate Ψ sites can be identified by some lab exploratory techniques, they are expensive and time-consuming (Carlile et al., 2014). As an increasing number of genomic and proteomic samples are produced (J. Yang et al., 2020), it is necessary to develop some effective and robust computational models to detect Ψ sites in RNA sequences.

Many machine learning algorithms have been introduced as fast, low-cost, and efficient alternative methods to identify Ψ sites. In 2015, Li et al. established the first computational model named PPUS to identify PUS-specific Ψ sites in Saccharomyces cerevisiae and Homo sapiens. The method used the nucleotides around Ψ as features for training a support vector machine (SVM) (Y. H. Li et al., 2015). Similarly, in 2016, Chen et al. developed an SVM classifier named iRNA-PseU using the occurrence frequencies and the chemical properties of the nucleotides as well as pseudo k-tuple nucleotide composition (PseKNC) as features in *Mus musculus*, *S. cerevisiae*, and *H. sapiens* (Chen et al., 2016). He et al., in 2018, proposed PseUI, in which five types of features, nucleotide composition (NC), dinucleotide composition (DC), pseudo dinucleotide composition (PseDNC), position-specific nucleotide composition (PSNP), and position-specific dinucleotide propensity (PSDP), were combined and a sequential forward selection method was applied to select the optimal feature subset for training SVM to predict Ψ sites in *M. musculus, S. cerevisiae*, and *H. sapiens* (J. He et al., 2018). In 2019, Liu et al. proposed an ensemble model, XG-PseU, based on eXtreme gradient boosting (XGBoost) using six types of features, including NC, dinucleotide composition (DNC), trinucleotide composition (TNC), nucleotide chemical property (NCP), nucleotide density (ND), and one-hot encoding (Liu et al., 2020). In 2020, Bi et al. proposed an integrated model based on a majority voting strategy, called EnsemPseU, which contained five machine learning methods SVM, XGBoost, Naive Bays (NB), k-nearest neighbor (KNN), and random forest (RF) (Bi et al., 2020). In short, the above machine learning methods in *H. sapiens*, *S. cerevisiae*, and *M. musculus* have the highest accuracy rates of 65.44%, 68.15%, and 72.03%, respectively. Although the performance of the above machine learning methods is reasonable, there is still a lot of room for improvement. With the emergence of deep learning methods, many prediction methods based on deep learning have been applied to the field of RNA and protein modification predictions (Huang et al., 2018; Long et al., 2018; Mostavi et al., 2018; Zhang and Hamada, 2018). The above predictors do not consider deep learning methods, which can extract deeper features to improve prediction performance (B. He et al., 2020; Liang et al., 2020).

**FIGURE 2:**
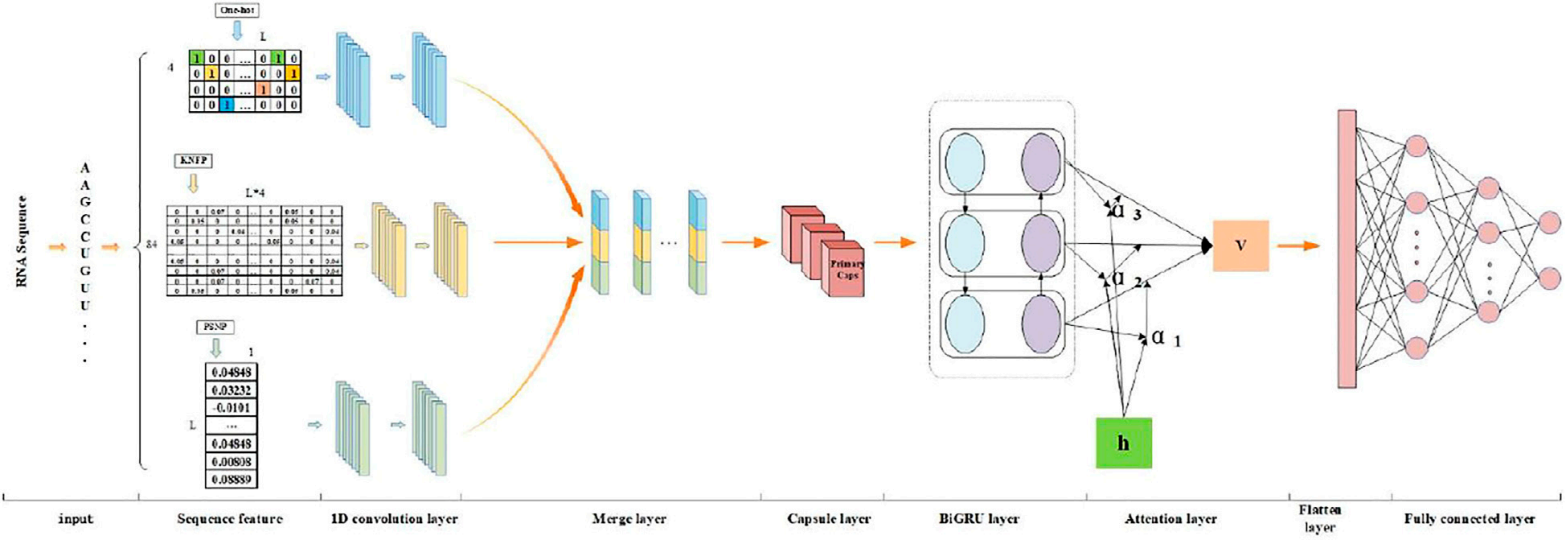
The flowchart of PseUdeep: We use the collected RNA sequences as the input of the model and the first use three encoding methods, one-hot encoding, KNFP, and PSNP, to extract RNA sequence features. Then, the three feature matrices are convoluted twice, and the results are stitched together. Finally, it is input into the capsule neural network and the BiGRU network with a self-attention mechanism and two fully connected layers for classification.

In this work, we propose a deep learning framework, PseUdeep, to identify Ψ sites of the three species *H. sapiens*, *S. cerevisiae*, and *M. musculus*. Compared with previous machine learning methods, our model applies three encoding methods, one-hot encoding, K-tuple nucleotide frequency pattern (KNFP) (Y. Yang et al., 2021), and PSNP (Dou et al., 2020) to extract RNA sequence features. Our model consists of a convolutional neural network (CNN), a capsule neural network, and a bidirectional gated recurrent unit (BiGRU) network with a self-attention mechanism (see Figure 2). Finally, we conduct a tenfold cross-validation test on the benchmark data set and an independent verification test on two independent data sets and compare the prediction results of our model with the results of the previous machine learning model; the accuracy of our model for *H. sapiens* increased by 1.55%, for *S. cerevisiae* by 4.58%, and for *M. musculus* by 0.42%.

## Methods

### Benchmark Data Sets

Chen et al. (2016) established data sets for computationally identifying Ψ sites in *H. sapiens*, *M. musculus*, and *S. cerevisiae* based on RMBase (Sun et al., 2016). With the update of RMBase, we use three training new data sets base on RMBase2.0 (Chen et al., 2015), which include NH_990 (*H. sapiens*), NM_944 (*M. musculus*), and NS_627 (*S. cerevisiae*), and the data sets built by Liu K. et al. (2020). In *H. sapiens* and *S. cerevisiae*, we also use the independent data sets H_200 and S_200, which are built by Chen et al. (2016) to evaluate the performance of the method.

In the NH_990 and NM_944 data sets, the length of the sequence is 21 nt. However, in the NS_627 data set, the length is31 nt. In the H_200 and S_200 data sets, the RNA sequence length is 21 and 31 nt, respectively. Table 1 shows the details of all data sets.

**TABLE 1:** The information on training data sets and independent testing data sets.

Species	The name of the datasets	The length of the RNA sequences (bp)	The number of positive samples	The number of negative samples
*H. sapiens*	NH-990 (training)	21	495	495
	H-200 (testing)	21	100	100
*S. cerevisiae*	NS-627 (training)	31	314	313
	S-200 (testing)	31	100	100
*M. musculus*	NM-944 (training)	21	472	472
	-	-	-	-

### Feature Extraction

Feature extraction is the basis of the algorithm. In our work, we consider three kinds of features: one-hot encoding, KNFP (Y. Yang et al., 2021), and PSNP (Dou et al., 2020).

#### One-Hot Encoding

Given an RNA sequence R,

$$R_{\phi }=\;N_{1}N_{2}\cdots N_{l}\text{,}$$ (1)

where $N_{j}\in \left\{A,C,G,U\right\}\left(j=1,2,\cdots ,l\right)$ represents the nucleotide at the jth position of the RNA segment R . We represent each nucleotide with a four-dimensional vector, that is, nucleotide G is represented as (1, 0, 0, 0),  C is (0, 1, 0, 0), U is (0, 0, 1, 0), and A  is (0, 0, 0, 1).

#### KNFP

The KNFP (Y. Yang et al., 2021) pattern represents the local contextual features at different levels. KNFP integrates various short-distance sequence order information and retains a large number of original sequence modes (Chen et al., 2015). We apply KNFP to extract local context features from RNA sequences. KNFP includes mononucleotide, dinucleotide, and trinucleotide composition. For an RNA sequence $R_{\phi }$ , the K-tuple nt composition can represent any RNA sequence as a $4^{\text{K}}$ dimensional vector:

$$\text{P}={\left[{\text{φ}}_{1},{\text{φ}}_{2},{\text{φ}}_{3},{\text{φ}}_{4},\ldots ,{\text{φ}}_{4^{\text{K}}}\right]}^{\text{T}}\text{,}$$ (2)

where $\phi_{u}\left(u=1,2,\cdots ,4^{\text{K}}\right)$ is the frequency of the uth  K  -tuple pattern in the RNA sequence, namely, the substring of the sequence contains K neighboring nt, and the symbol T represents the transpose operator, so it has l−K+1 overlapping segments for every RNA sequence   R with length  l  , and each segment is encoded as a one-hot vector with dimension $4^{\text{K}}$ . The frequency pattern matrix $m_{\text{K}}\;\varepsilon {\text{R}}^{\left(l-\text{K}+1\right)\text{*}4^{\text{K}}}$ is generated for each type of K-tuple nt composition. To facilitate subsequent processing, we fill the shorter part with zeros. By combining different K-tuples $\text{ M}=\left\{{\text{m}}_{1},{\text{m}}_{2},{\text{m}}_{3}\right\}$ with K=1,2,3 , the feature of each position in the sequence is connected in one dimension of size d=64 .Compared with the traditional one-hot encoding, KNFP effectively compensates for the shortcomings of information insufficiency.

#### PSNP

PSNP (Dou et al., 2020) is an effective nucleotide encoding method, which has been successfully applied to the identification of many functional sites in biological sequences (W. He et al., 2018; W. He et al., 2018; G. Q. Li et al., 2016; Zhu et al., 2019). In this method, location-specific information can be represented by calculating the differences in nucleotide frequency at a specific location between positive and negative RNA samples. Considering an RNA sequence $R_{\phi }=\;N_{1}N_{2}\cdots N_{l}$ , the PSNP matrix can be written as a 4×l  -dimensional vector.

First, we calculate the frequency of occurrence for four nucleotides, respectively, from bath positive and negative samples at the *j*th position. In this way, we obtain two 4×l  position-specific occurrence frequency matrixes, namely, $Z^{+}$ and $\;Z^{-}$ , of which $Z^{+}$ is obtained from all positive samples and $Z^{-}$ from all negative samples. We define the location-specific nucleotide propensity matrix, represented by $\;Z_{PSNP}$ , as shown below:

$$Z_{PSNP}=\left[Z_{1},Z_{2,\cdots }Z_{l}\right]=[\begin{array}{cccc} Z_{1,1} & Z_{1,2} & \cdots & Z_{1,l}\\ Z_{2,1} & Z_{2,2} & \cdots & Z_{2,l}\\ Z_{3,1} & Z_{3,2} & \cdots & Z_{3,l}\\ Z_{4,1} & Z_{4,2} & \cdots & Z_{4,l} \end{array}]\text{,}$$ (3)

where $Z_{i,j}=Z_{i,j}^{+}-Z_{i,j}^{-}$ gives the difference of frequencies of the ith nucleotide at the jth  position between positive and negative samples.

### Deep Learning Architecture of PseUdeep

For each input sequence, we use three feature extraction (one-hot encoding, KNFP, and PSNP) methods to form three feature matrices. For each feature matrix, a pair of 1-D CNNs are used. The first layer of each feature matrix has a filter size of 11 and a kernel size of 7. Similarly, the second 1[D CNN layer for each feature matrix has a filter size of 11 and a kernel size of 3. Two convolution layers are used to capture features from three feature matrices; all layers had a “Relu” activation function. The three convolution results are spliced together and fed into the capsule network with 14 capsules for vector convolution, and the output of the capsule network is put into the BiGRU neural network with an attention mechanism; the final feature is concatenated and fed into two dense layers to obtain the prediction results. Bayesian optimization is used to select the best performance of the hyperparameters. The adjusted parameters are the number of filters, the filter size, and epoch. To prevent the model from overfitting, the dropout algorithm with a probability of 0.5 is also used. A binary cross-entropy is used as a loss function with an early stop patience of 20. The batch size is 32, and the number of epochs is set to 200. For the stochastic gradient descent method, the Adam optimization algorithm is selected here. The total number of trainable parameters in the network is 165,365. The entire program is done in Python 3.6.

#### CNNs

CNNs are widely used in the fields of artificial intelligence, such as machine learning, speech recognition, document analysis, language detection, and image recognition.

#### Capsule Neural Networks

Capsule neural networks, first proposed by Hinton et al., provide a unique and powerful deep learning component to better simulate the various relationships represented inside the neural network. Because capsule neural networks can collect location information, they can learn a small amount of data to get good predicted results. In the data sets we collected, the amount of RNA data is small, and the length of RNA sequences is small, so to study the hierarchical relationship of local features, capsule neural networks are used in this paper.

#### BiGRU Networks and Attention Mechanism

BiGRU networks are used to extract the deep features of the sequences because BiGRU networks can be regarded as two unidirectional GRUs. An attention mechanism in a deep neural network is also an important part. The attention mechanism is remarkable in serialized data, such as speech recognition, machine translation, and part of speech taming, which has also been widely used in much bioinformatics research and achieved excellent performance.

### Cross-Validation and Independent Testing

Because the K -fold ( K = 5 or 10) cross-validation (Dezman et al., 2017; G. Q.; Li et al., 2016; Vučković et al., 2016) is widely used to evaluate models, we apply a tenfold cross-validation test to measure model performance in NH_990, NM_944, and NS_627, in which a data set can be divided into 10 mutually exclusive folds, one fold is reserved for testing, whereas the remaining nine folds are used for training purposes. To verify the stability of the models more objectively, the proposed models are tested on two independent data sets H_200 and S_200.

### Performance Evaluations

To measure the performance of our model, we use four statistical parameters, sensitivity (Sn), specificity (Sp), accuracy (Acc), and Matthew’s correlation coefficient (MCC), which are used in a series of studies to evaluate the effectiveness of predictors. These parameters are defined as follows:

$$Sn=1-\frac{N_{-}^{+}}{N^{+}}\text{,}$$ (4)

$$Sp=1-\;\frac{N_{+}^{-}}{N^{-}}\text{,}$$ (5)

$$Acc=1-\frac{N_{-}^{+}+N_{+}^{-}}{N^{+}+N^{-}}\text{,}$$ (6)

$$MCC=\;\frac{1-\frac{N_{-}^{+}+N_{+}^{-}}{N^{+}+N^{-}}}{\sqrt{\left(1+\frac{N_{+}^{-}-N_{-}^{+}}{N^{+}}\right)\left(1+\frac{N_{-}^{+}-N_{+}^{-}}{N^{-}}\right)}}\text{,}$$ (7)

where $N^{+},N^{-}$ indicate the number of positive and negative sequences, respectively; $N_{-}^{+}$ represents the number of positive RNA samples that are incorrectly predicted as negative RNA samples; and ${\text{N}}_{+}^{-}$ represents the number of negative RNA samples that are incorrectly predicted as positive RNA samples. In addition, the graph of the ROC (Fawcett, 2006) is also widely used to intuitively display the performance. Then, the AUC can be obtained to objectively evaluate performances of the proposed model.

## Results

### Model Selection

To select a more effective model, in each data set, we first compare four models’ performances based on two feature extraction methods, one-hot encoding and KNFP (results are shown in Supplementary Tables S1, S2). These models are constructed by gradually adding different types of layers based on two 1-D convolution layers, a BIGRU network, and a two fully connected layers network. The four models are shown below:

1) CNN: The network consists of two layers of 1-D convolution, a BIGRU network, and a two fully connected layers network as described above. The input matrices are the one-hot encoding and KNFP features extracted from the RNA sequences.
2) CNN + Capsule: The model adds a capsule layer after the BiGRU layer on the basis of the CNN model.
3) CNN + Attention: The model adds a self-attention mechanism layer before the BiGRU layer based on the CNN model.
4) CNN + Capsule + Attention: The model adds a capsule layer based on the CNN + Attention model; on the basis of the above four models, we add PSNP features and compare the performance of the four new models (see Tables 2, 3). In summary, our PseUdeep model (CNN + Capsule + Attention model on three feature extraction methods) is superior to the others.

**TABLE 2:** Tenfold cross-validation performance comparison of four models based on three feature extraction methods on three benchmark data sets.

Data sets	Models	Accuracy (%)	Sensitivity (%)	Specificity (%)	MCC	AUC
NH_990	CNN	**67.96**	68.09	67.86	**0.36**	0.737
	CNN + Capsule	66.02	63.83	67.86	0.32	0.742
	CNN + Attention	66.02	46.81	**82.14**	0.31	0.745
	PseUdeep (CNN+	66.99	**74.47**	60.71	0.35	**0.746**
	+Capsule + Attention)					
NS_627	CNN	69.71	70.59	68.75	0.39	0.728
	CNN + Capsule	68.18	61.76	75.00	0.37	0.735
	CNN + Attention	69.71	**76.47**	68.75	0.40	0.734
	PseUdeep (CNN	**72.73**	61.75	**78.13**	**0.45**	**0.737**
	+Capsule + Attention)					
NM_944	CNN	70.41	57.78	**86.79**	0.41	0.741
	CNN + Capsule	69.39	73.34	66.04	0.39	0.750
	CNN + Attention	70.41	57.78	81.13	0.41	0.751
	PseUdeep (CNN	**72.45**	**66.70**	77.36	**0.44**	**0.756**
	+Capsule + Attention)					

The bold value is the value with the best effect in the corresponding evaluation index.

**TABLE 3:** Performance comparison of four models based on three feature extraction methods on independent testing data sets.

Testing data sets	Models	Accuracy (%)	Sensitivity (%)	Specificity (%)	MCC	AUC
H_200	CNN	65.69	68.63	62.75	0.31	0.691
	CNN + Capsule	62.25	63.73	60.78	0.25	0.696
	CNN + Attention	65.19	52.94	**77.45**	0.31	0.692
	PseUdeep (CNN	**66.18**	**73.53**	58.82	**0.33**	**0.720**
	+Capsule + Attention)					
S_200	CNN	**82.35**	**86.27**	78.43	0.65	0.899
	CNN + Capsule	80.88	77.45	84.31	0.62	0.908
	CNN + Attention	79.91	83.34	76.47	0.59	0.899
	PseUdeep (CNN	80.88	77.45	**84.31**	**0.65**	**0.909**
	+Capsule + Attention)					

The bold value is the value with the best effect in the corresponding evaluation index.

### Performance of a Single Type of Feature

We also evaluate our model (CNN + Capsule + Attention) with only one kind of feature. Table 4 shows the comparison of performance in the tenfold cross-validation on benchmark data sets. It follows that the ACC values and AUC values of PSNP in three species, *H. sapiens*, *M. muscles*, and *S. cerevisiae*, are much higher than those of the other two characteristics. The ACC value of PSNP is increased by 11.11%, 15.6%, and 16.68%, respectively, compared with other characteristics, the AUC value increased by 0.074, 0.199, and 0.115, respectively. PSNP provides a great possibility to improve the model performance in identifying Ψ sites.

**TABLE 4:** The model performance with a single type of feature.

Benchmark data sets	Models	Accuracy (%)	Sensitivity (%)	Specificity (%)	MCC	AUC
NH_990	one-hot	55.56	40	68.51	0.08	0.592
	PSNP	**66.67**	62.22	**70.37**	**0.32**	**0.666**
	KNFP	63.63	**80**	50	0.31	0.658
NS-627	one-hot	53.03	26.47	**81.25**	0.09	0.634
	PSNP	**69.71**	61.75	78.13	**0.40**	**0.734**
	KNFP	66.67	**64.71**	68.75	0.33	0.619
NM-944	one-hot	58.16	35.55	77.35	0.14	0.547
	PSNP	**71.42**	57.77	**83.01**	**0.42**	**0.746**
	KNFP	56.12	**62.22**	50.94	0.13	0.580

The bold value is the value with the best effect in the corresponding evaluation index.

### Comparison with State-of-the-Art Methods

**FIGURE 3:**
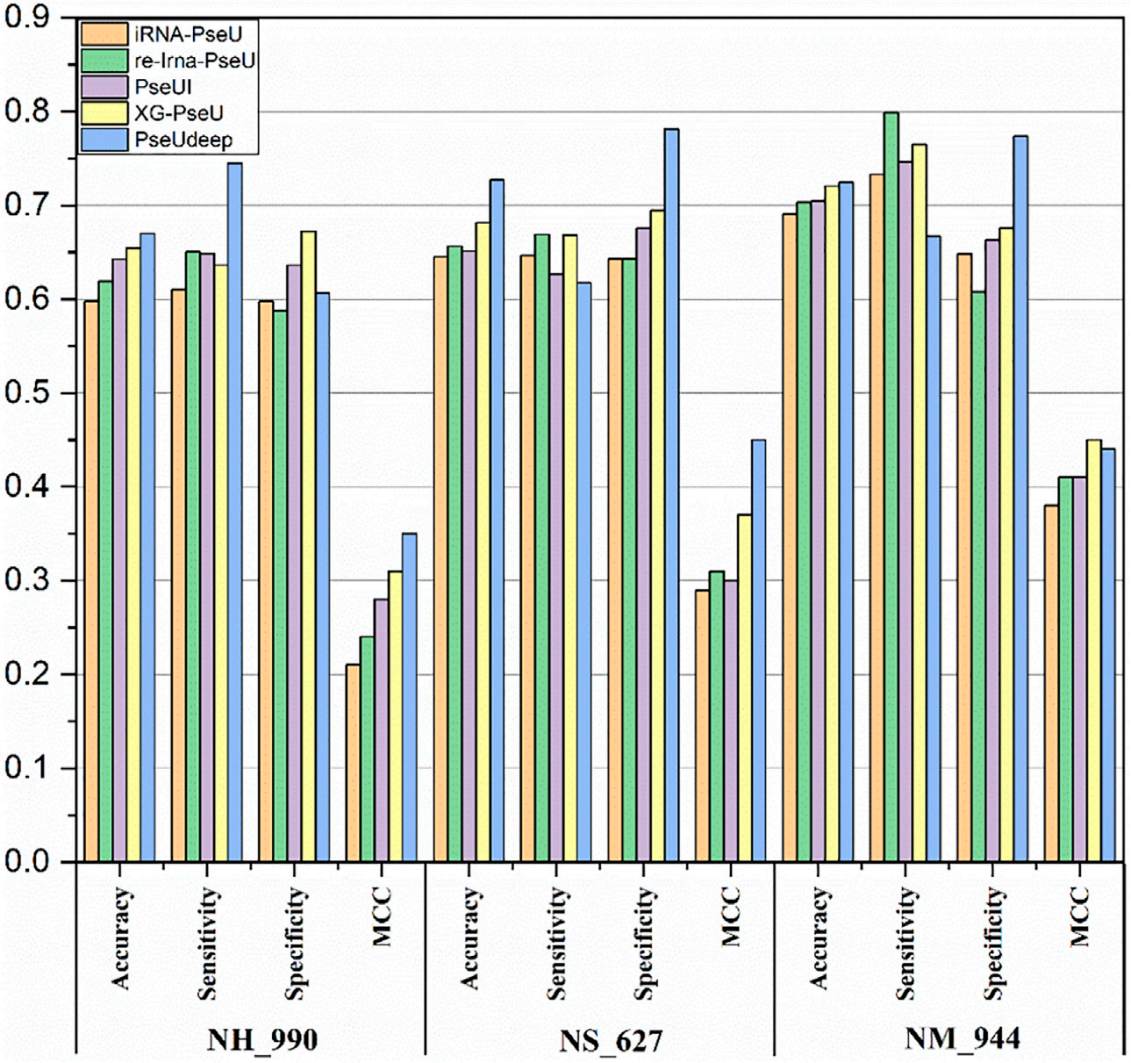
The success rates of the PseUdeep and baseline methods on three training data sets.

**FIGURE 4:**
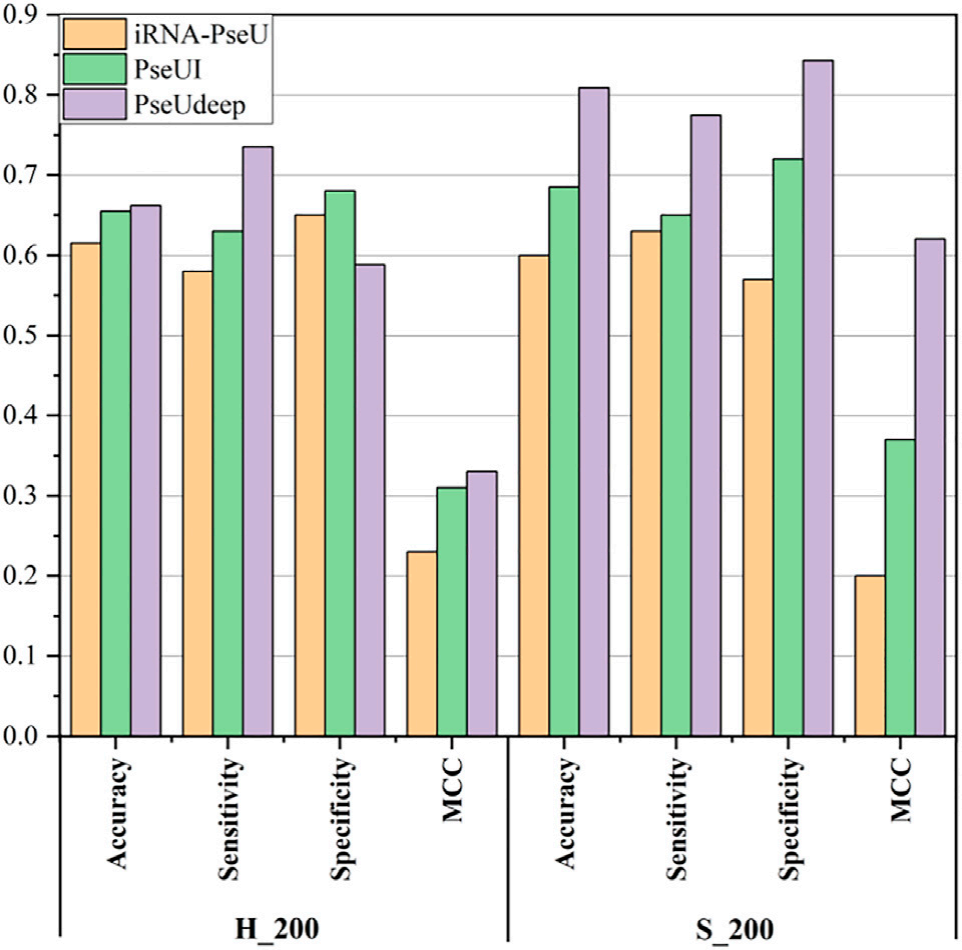
The success rates of the PseUdeep and baseline methods on independent data sets.

**FIGURE 5:**
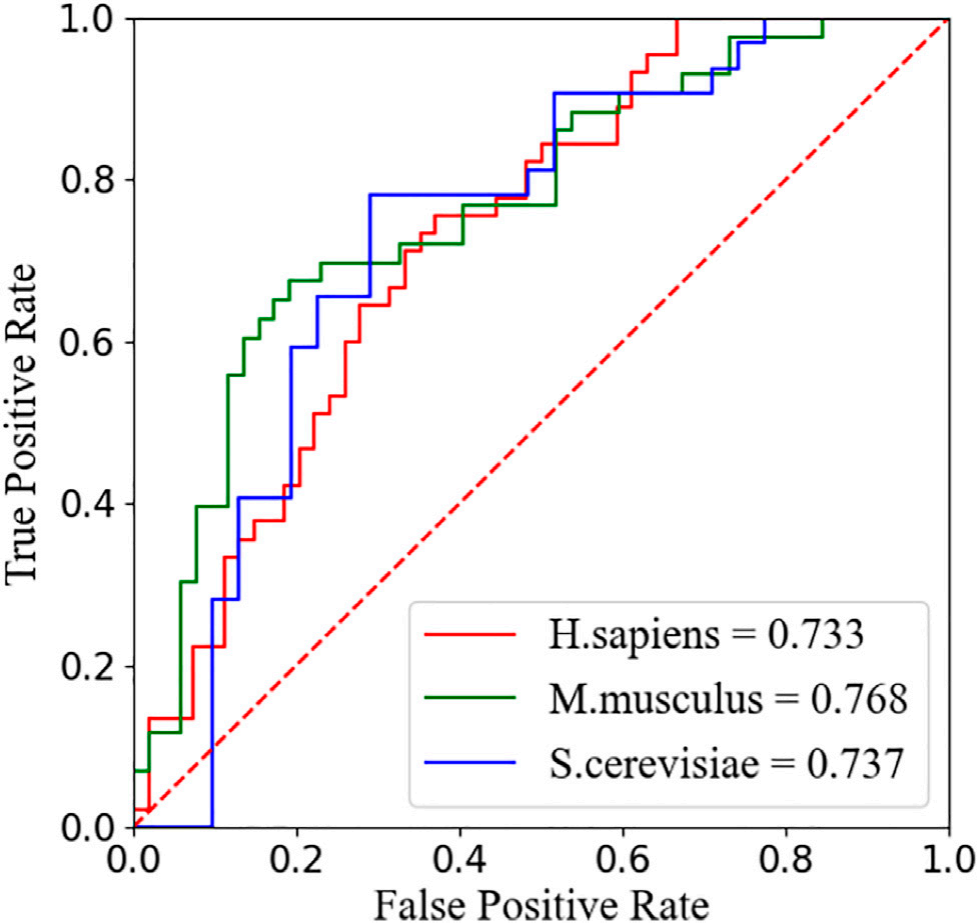
The ROC curves of PseUdeep for *H. sapiens*, *S. cerevisiae*, and *M. musculus*, respectively.

We compare our model PseUdeep with other state-of-the-art machine learning predictors published recently to evaluate the identification ability of Ψ sites. In benchmark data sets with tenfold cross-validation and independent testing, the results obtained by PseUdeep and other predictors are listed in Tables 5, 6 and Figures 3, 4; the ROC curves of PseUdeep are shown in Figure 5. The accuracy of the PseUdeep model in NH_990, NS_627, and NM_944 is increased by 1.55%, 4.58%, and 0.32%. In addition, the performance of PseUdeep on independent data sets compared with iRNA-Pse and PseUI is shown in Table 6 and Figure 4. It can be observed that the accuracy of the PseUdeep model in H_200 and S_200 is increased by 0.68% and 12.38%, respectively.

**TABLE 5:** A comparison of PseUdeep with other models on three benchmark data sets.

Training data set	Models	Accuracy (%)	Sensitivity (%)	Specificity (%)	MCC	AUC
NH_990	iRNA-PseU	59.80	61.01	59.80	0.21	0.61
	re-Irna-PseU	61.92	65.05	58.79	0.24	0.65
	PseUI	64.24	64.85	63.64	0.28	0.68
	XG-PseU	65.44	63.64	**67.24**	0.31	0.70
	PseUdeep	**66.99**	**74.47**	60.71	**0.35**	**0.74**
NS-627	iRNA-PseU	64.49	64.65	64.33	0.29	**0.81**
	re-Irna-PseU	65.61	**66.88**	64.33	0.31	0.69
	PseUI	65.13	62.72	67.52	0.30	0.69
	XG-PseU	68.15	66.84	69.45	0.37	0.74
	PseUdeep	**72.73**	61.75	**78.13**	**0.45**	0.74
NM-944	iRNA-PseU	69.07	73.31	64.83	0.38	0.75
	re-Irna-PseU	70.34	**79.87**	60.81	0.41	0.75
	PseUI	70.44	74.58	66.31	0.41	0.77
	XG-PseU	72.03	76.48	67.57	0.45	0.77
	PseUdeep	**72.45**	66.7	**77.36**	**0.44**	**0.77**

The bold value is the value with the best effect in the corresponding evaluation index.

**TABLE 6:** A comparison of PseUdeep with other models on independent data sets.

Testing dataset	Models	Accuracy (%)	Sensitivity (%)	Specificity (%)	MCC	AUC
H_200	iRNA-PseU	61.5	58	65	0.23	/
	PseUI	65.5	63	**68**	0.31	/
	PseUdeep	**66.18**	**73.53**	58.82	**0.33**	**0.720**
S_200	iRNA-PseU	60	63	57	0.2	/
	PseUI	68.5	65	72	0.37	/
	PseUdeep	**80.88**	**77.45**	**84.31**	**0.62**	**0.909**

The bold value is the value with the best effect in the corresponding evaluation index.

We summarize and compare our model with other state-of-the-art models in terms of feature extraction, number of features, and classifiers as shown in Table 7. Among them, our model PseUdeep does not further feature selection, and the feature dimension is only 109, 109, and 119 in *H. sapiens*, *M. musculus*, and *S. cerevisiae*, respectively, and our model gets the highest accuracy of the prediction.

**TABLE 7:** Five methods to identify Ψ sites are summarized in all aspects.

Method	Feature extraction	Number of features	Classifiers
iRNA-PseU	PseKNC	$\{\begin{array}{c} H.\;sapiens\;\;\;\;\;\;\;\;\;\;\;\;\;\;\;\;84\\ M.\;musculus\;\;\;\;\;\;\;\;\;\;\;\;84\\ S.\;cerevisiae\;\;\;\;\;\;\;\;\;\;\;\;124 \end{array}$	SVM
PseUI	NC + DC + pseDNC + PSNP + PSDP	$\{\begin{array}{c} H.\;sapiens\;\;\;\;\;\;\;\;\;\;\;\;\;\;\;\;1045\\ M.\;musculus\;\;\;\;\;\;\;\;\;\;\;\;1045\\ S.\;cerevisiae\;\;\;\;\;\;\;\;\;\;\;\;1526 \end{array}$	SVM
XG-PseU	One-hot + TNC + NCP + ND + DNC	$\{\begin{array}{c} H.\;sapiens\;\;\;\;\;\;\;\;\;\;\;\;\;\;\;\;1848\\ M.\;musculus\;\;\;\;\;\;\;\;\;\;\;\;1848\\ S.\;cerevisiae\;\;\;\;\;\;\;\;\;\;\;\;2728 \end{array}$	XGBoost
EnsemPseU	Kmer + Binary + ENAC + NCP + ND	>1700	SVM + XGBoost + NB + KNN + RF
PseUdeep	One-hot + PSNP + KNFP	$\{\begin{array}{c} H.\;sapiens\;\;\;\;\;\;\;\;\;\;\;\;\;\;\;\;109\\ M.\;musculus\;\;\;\;\;\;\;\;\;\;\;\;109\\ S.\;cerevisiae\;\;\;\;\;\;\;\;\;\;\;\;119 \end{array}$	Deep learning network

## Conclusion

In this study, we propose a model, PseUdeep, which can effectively identify Ψ sites in RNA sequences. To get better prediction performance, we also train a combination of three features in a simple model and then gradually add different types of layers to obtain better performance. In addition, we compare our model with other models through tenfold cross-validation and independent testing, and the results show that PseUdeep is more accurate and stable. Finally, we evaluate and compare the performance of the three features used in this study and find that PSNP shows the best effect.

## Data Availability

The original contributions presented in the study are included in the article/Supplementary Material, further inquiries can be directed to the corresponding author.
