# Supplementary material for: PseUdeep: RNA Pseudouridine Site Identification with Deep Learning Algorithm
Source: Front Genet. 2021 Nov 18;12:773882. doi: 10.3389/fgene.2021.773882 (PMC8637112; doi:10.3389/fgene.2021.773882)
Supplement: Supplementary file 1 [file Table1.DOCX]

**Table S1**: 10-fold cross-validation performance comparison of four models based on KNFP+one-hot feature extraction methods on independent testing datasets

| Testing Dataset | Models | Accuracy (%) | Sensitivity (%) | Specificity (%) | MCC | AUC |
| --- | --- | --- | --- | --- | --- | --- |
| H_200 | CNN | **66.66** | 67.16 | **71.56** | **0.33** | **0.737** |
|  | CNN+Capsule | 63.72 | 63.72 | 63.72 | 0.27 | 0,661 |
|  | CNN+Attention | 59.31 | **81.37** | 37.25 | 0.21 | 0.627 |
|  | CNN+Capsule+Attention | 54.91 | 67.65 | 42.17 | 0.11 | 0.634 |
| S_200 | CNN | **62.25** | **47.07** | 77.45 | **0.25** | **0.692** |
|  | CNN+Capsule | 55.39 | 21.57 | **89.24** | 0.14 | 0.549 |
|  | CNN+Attention | 56.86 | 24.51 | 89.22 | 0.18 | 0.644 |
|  | CNN+Capsule+Attention | 56.86 | 30.39 | 83.33 | 0.16 | 0.574 |

**Table S2**: Performance comparison of four models based on KNFP+one-hot feature extraction methods three benchmark datasets

| Training Dataset | Models | Accuracy (%) | Sensitivity (%) | Specificity (%) | MCC | AUC |
| --- | --- | --- | --- | --- | --- | --- |
| NH_990 | CNN | 61.61 | 46.80 | **73.21** | 0.21 | **0.665** |
|  | CNN+Capsule | 60.19 | 48.93 | 69.64 | 0.19 | 0.655 |
|  | CNN+Attention | 53.39 | 78.72 | 32.14 | 0.12 | 0.561 |
|  | CNN+Capsule+Attention | **62.13** | **82.97** | 44.64 | **0.29** | 0.637 |
| NS_627 | CNN | **60.01** | **55.88** | 65.62 | **0.21** | **0.681** |
|  | CNN+Capsule | 56.06 | 32.35 | **81.25** | 0.16 | 0.671 |
|  | CNN+Attention | 56.06 | 35.29 | 78.12 | 0.14 | 0.629 |
|  | CNN+Capsule+Attention | 56.06 | 41.17 | 71.87 | 0.13 | 0.607 |
| NM_944 | CNN | **63.26** | 46.67 | **77.35** | **0.25** | **0.656** |
|  | CNN+Capsule | 59.18 | 57.78 | 60.38 | 0.18 | 0.619 |
|  | CNN+Attention | 56.12 | 46.67 | 64.15 | 0.11 | 0.621 |
|  | CNN+Capsule+Attention | 48.97 | **77.77** | 24.53 | 0.02 | 0.603 |
